# An adapted imaginal exposure approach to traditional methods used within trauma-focused cognitive behavioural therapy, trialled with a veteran population

**DOI:** 10.1017/S1754470X16000052

**Published:** 2016-06-01

**Authors:** Manveer Kaur, Dominic Murphy, Kirsten V. Smith

**Affiliations:** 1 Combat Stress, Tyrwhitt House, Leatherhead, Surrey, UK; 2 King’s Centre for Military Health Research, King’s College London, London, UK; 3 Oxford Centre for Anxiety Disorders and Trauma, Department of Experimental Psychology, Oxford, UK

**Keywords:** Dissociation, intrusions, memory fragmentation, PTSD, traumatic stress

## Abstract

Trauma-focused cognitive behavioural therapy (TF-CBT) is beneficial for individuals with post-traumatic stress disorder (PTSD); however, a subset of clients struggle to engage with traditional methods, due to high levels of avoidance and dissociation. This paper aims to describe an adapted approach to imaginal reliving and prolonged exposure, to facilitate subsequent cognitive updating. The paper demonstrates the technique with veterans, who are a client group that may struggle with some aspects of traditionally implemented TF-CBT. Two case studies are described, both with PTSD symptoms stemming from traumatic military experiences. An adapted exposure technique is utilized to address the barriers of high dissociation, poor access to trauma-related cognitions and fixed intrusive imagery. The approach involved three stages: (1) reliving the trauma outdoors, (2) manipulating the perspectives of the imagery, and (3) restructuring the narrative with new perspectives. Both clients showed decreased dissociation and improved toleration of their traumatic imagery. Improvement of PTSD symptoms and quality-of-life functioning was observed for both clients on objective measures. Adapting TF-CBT to have a stronger emphasis on grounding and allocentric processing may be helpful for a subset of patients with PTSD that present with high levels of dissociation and avoidance. Further research and investigation into alternative populations is needed.

## Introduction

Evidence-based treatments of post-traumatic stress disorder (PTSD) include trauma-focused cognitive behavioural therapy (TF-CBT; NICE, 2005). A central component of this approach is the imaginal reliving of intrusive memories (Ehlers & Clark, 2000; Grey *et al.* 2001). Associated distressing cognitions that emerge can then be modified (Ehlers & Clark, 2000), all of which aims to result in a more contextualized, organized and coherent memory of the trauma that is less prone to involuntary retrieval. Clinicians working within a TF-CBT framework may also draw upon Foa’s (2011) model of repeated exposure to stimuli to reduce emotional responding, through imaginal reliving. A recent study has suggested that it may be possible to facilitate reliving by encouraging allocentric (viewpoint-independent) processing (Smith *et al.* 2015). This is achieved by purposefully manipulating the viewpoint of the trauma memory in imagery (Brewin *et al.* 2010), a process by which the systems in the brain responsible for contextualizing a trauma memory and reducing emotionally salient intrusions are activated (King *et al.* 2002). This process aims to connect fragmented traumatic images with trauma cognitions and emotions in a spatially rich and temporally fluid memory. In this paper we discuss an adapted approach to support clients with fragmented trauma memories that has been designed to support allocentric spatial processing.

Recent data has suggested that TF-CBT can be beneficial for veterans (Murphy *et al.* 2015); however, as a client group research also suggests they have poorer outcomes than non-veterans (Bradley *et al.* 2005; Bisson *et al.* 2007). A recent study of the factors that predict treatment response in veterans reported that high levels of pre-treatment dissociation were associated with poorer post-treatment outcomes (D. Murphy & W. Bussutil, unpublished data). Therefore techniques that allow dissociation to be managed and help contextualize memories would be helpful.

The aim of this paper is to highlight useful adaptations to the imaginal reliving and prolonged exposure elements of interventions from cognitive and neurobiological models of trauma-focused therapy (Ehlers & Clark, 2000; Foa, 2011), to support greater toleration of dissociation and processing of the trauma memories. This approach can be a helpful adjunct to facilitate cognitive reappraisal strategies such as imagery rescripting (Hackmann, 2011) and compassion-focused approaches (Gilbert, 2010) which are currently well used within specialist traumatic stress services. This approach aims to be useful where clients have struggled with traditional methods of accessing cognitions, imaginal reliving and grounding techniques in the therapy room alone, and for those who find contextualization of a fragmented image challenging.

This paper seeks to extend the evidence base by describing two cases in which clients were encouraged to describe their traumatic experiences outdoors to manage dissociation levels, and to gradually build up a spatially dynamic cognitive map of the traumatic event that contained more allocentric frames of reference. Utility of this approach, theoretical underpinnings and further research will be discussed.

## Case reports

### Case 1

Mr C was a single man in his 60s. He had served in the UK military police during the Northern Ireland peacekeeping operations in the 1960s. During his service he attended the aftermath of an explosion that had killed several civilians, including a child. Afterwards he experienced an intrusive fragmented image of the scene in his right peripheral field of vision. At assessment he reported experiencing the image every day but avoided focusing on it. He had not spoken about the event to anyone in his life prior to the start of therapy. He identified that his mood state fluctuated between detachment and feeling overwhelmed by anxiety symptoms when confronted by a reminder of the event (e.g. a news item about Northern Ireland). This could lead him to dissociate and therefore he avoided such triggers. He also experienced periods of depression and had previously used alcohol to cope with his difficulties. His avoidance of the trauma memory maintained his PTSD symptoms (Ehlers & Clark, 2000). He reported he had not received any previous psychological therapy.

Mr C presented as avoidant of engaging in TF-CBT, and utilized several therapy sessions to discuss his concerns. He identified the appraisal as ‘I won’t be able to cope’. Mr C attempted to use multiple grounding strategies in the therapy room, combining olfactory strategies with standing up and holding an object; however, he either presented as matter of fact and detached from any emotion or unable to tolerate ‘*in vivo*’ reliving that involved closing his eyes, claiming he felt overwhelmed by physical panic symptoms and he began to dissociate. In both reliving sessions he could not gain access to his peri-traumatic cognitions.

### Case 2

Mr P was a married man in his 40s with one child. He had served in the UK army and was deployed to the Bosnian conflict in the mid-1990s. During his deployment he was stationed at a morgue that had been set up to aid the identification of bodies recovered from a mass burial site. He described how the morgue contained a large number of bodies in various states of decomposition, with varying degrees of physical trauma. He reported re-experiencing fragmented intrusive images of the morgue and daily emotionally distressing nightmares of which he could not recall the content. He experienced high levels of anxiety that triggered his dissociative symptoms. These appeared to function as a coping strategy to avoid distressing emotions connected to his memory of the morgue. Other avoidance symptoms included feeling detached from people around him and feeling emotionally numb. Mr P avoided family occasions and busy places which could trigger his hyper-arousal symptoms and had never spoken to anyone about his experiences. He reported coping historically through the use of alcohol to block out his emotions. His avoidance behaviours had maintained his PTSD symptoms.

At the start of therapy sessions, Mr P was reluctant to talk about his past experiences stating he only felt able to discuss vague details about his intrusive memories. Mr P attempted to use physical objects such as stones and leaves, or his e-cigarette, as grounding strategies in the clinic room. However, he also reported feeling unable to tolerate any emotions connected to the events and found it difficult to identify peri-traumatic cognitions. Mr P appeared to experience intrusions of one particular fragmented image and could not recall any other contextual information from before or after this moment.

In both cases Mr C and Mr P experienced difficulties in engaging with traditional methods of imaginal reliving and prolonged exposure, due to high levels of avoidance and low thresholds for dissociation, and subsequently had difficulties accessing their cognitions for updating. For both clients the prior use of multiple grounding methods in the therapy room were unsuccessful. They experienced their traumatic memories as fragmented images with a sense of ‘nowness’ (Birrer *et al.* 2007), which indicated a distinct lack of contextualization.

An adaptive approach was then trialled to address these hindering factors. This involved three stages: (1) walking the client through the imaginal scene outdoors to address dissociation, (2) viewing the imaginal scene from multiple perspectives to facilitate contextualization of the memory and (3) identifying and reappraising the cognitions with frequently used approaches in trauma-focused therapy.

## Context of the study

Both clients were attending a 6-week residential programme for the treatment of PTSD, which involved attending psychoeducation and skill-based groups, alongside 15–18 sessions of individual TF-CBT. Further details about this intervention and treatment outcomes have been published elsewhere (Murphy *et al.* 2015). The approach was utilized during their individual therapy sessions in the grounds of the treatment centre, with the clients attending 2–3 sessions per week. Additional sessions beyond the usual course of treatment were not required in order to offer this adapted approach to intervention.

Prior to attending the programme, both veterans had completed previous short-term admissions, which focused on attending stabilization groups and provided an opportunity to socialize to the therapy environment. This also supported the reduction of stigma and normalization of symptoms with other peers. Both clients were required to maintain abstinence from substances during the programme, and therefore prior work around substance misuse had been addressed beforehand. The adapted reliving techniques were implemented only after a comprehensive collaborative formulation was developed that indicated the approach’s utility in these particular cases. Standardized outcome measures were administered at the start and end of the programme, and at 6-week and 6-month review points, which are discussed later.

Prior preparation was undertaken for outdoor imaginal reliving. The rationale of this approach was considered with clients beforehand and will be discussed further. Confidentiality was addressed by utilizing residential grounds, which were connected to the treatment centre but sufficiently distant from other individuals to allow privacy during the process. A risk assessment was undertaken by the therapists, including consideration of risk of harm to self and/or others, and plans of action discussed in the case of dissociation. The clinical team indoors were informed of the session’s whereabouts prior to and post-session.

## Treatment approach

### Walking through the narrative

By reliving the imagined scene outdoors, this initial stage aimed to provide additional sensory grounding elements that were lacking in the clinic rooms. In doing so, we hoped to address the problem of clients easily dissociating while trying to discuss their trauma memories, and allow for access to their peri-traumatic cognitions. Second, we encouraged the clients to construct a 3D cognitive map of the spatial scene of the trauma, within which they could move around freely and focus on different elements. Conducting this outdoors as opposed to the clinic room allowed for multiple, sensory grounding elements, freedom of movement and space to create a rich construction of the scene.

Both clients appeared to benefit from a focus on multiple sensations in the outdoors to help ground themselves. For example, Mr C was encouraged to notice the sensation of cold air against his skin during his movement, and the feeling of the grass beneath his bare feet. Mr P used visual landmarks of bushes and trees to stay grounded in the present, as well as using auditory elements (e.g. the sound of the wind and local traffic). This prevented both clients from dissociating while ‘reliving’ their traumatic events, and in doing so allowed them to enter a more ‘optimal zone’ for processing the memory. Both clients were also able to gain greater access to their peri-traumatic emotions and cognitions through this adapted ‘*in vivo*’ approach. For example, Mr P could now recall the peri-traumatic cognition ‘this is really happening’, which was previously inaccessible as he had dissociated at the time of the trauma. Both Mr C and Mr P expressed peri-traumatic feelings of horror and guilt, and related this to the cognitions such as ‘It’s too awful to see’ and ‘I should have done something’.

They then visualized the scene outdoors, to help further process the image. Over several sessions both clients noted greater toleration of anxiety over this increased exposure. Mr P was able to construct a richer description of the morgue scene, both in terms of the detail and positioning of the bodies and details of the scene around the bodies. Mr C was able to tolerate focusing on the imaginal scene for increasing periods of time before moving on, using visual landmarks for reference which could then also be used to discriminate and ground himself (e.g. using two bushes to represent bodies in the explosion and the space in between to represent the wreckage from the explosion). At this stage, both clients began to move away from the fragmented nature of the images into a more fluid event. Mr P was able to do this with increased detail of the environment of the morgue and Mr C was able to ‘walk through’ the events prior to and after the explosion, placing them in a broader context. Increasing their visuo-spatial perspectives of the scene in the next stage further supported this process.

### Manipulating the perspectives

The aim of this stage was to encourage the client to utilize the imaginal trauma scene, and begin to view it from different visual perspectives and angles, to facilitate allocentric processing. Through this kinetic exploration, we aimed for the scene to move on from a frozen image to a fluid 3D memory with more allocentric frames of reference, and increase the possibility of alternative cognitions to develop. Both clients continued to utilize the benefits of the sensory grounding elements outdoors while doing so.

In this phase clients were encouraged to move around the scene and focus on the spatial relationships of elements within the scene as they moved. Mr P did so by imagining the morgue from different angles in the room (e.g. how it looked from the left to right as opposed to his original position). For example, his intrusive image of the body had been focused on the decapitation neck wound from his original perspective. However, by changing perspective Mr P was able to view alternative information about the body (e.g. greater view and attention to the man’s clothes and that he wore a wedding ring), theoretically facilitating allocentric processing of the scene and the trauma memory. This began to allow for some connection to alternative emotions of sadness and accessing of cognitions (e.g. ‘this man had a family’ and ‘this man mattered’). He also used a future perspective to consider where the body would be now, and ground himself as a reminder that ‘it is no longer here’.

In the case of Mr C, he began by viewing the scene of the explosion from different angles, some of which were less distressing to him than the original perspective of the scene (e.g. to walk around the back of the explosion aftermath where he could not see the bodies, but could see the wreckage in more detail). He was then able to draw from imagery rescripting and compassion-focused methods, by viewing the scene as an observer from the future. To do so, he imagined ‘zooming’ out of the scene to observe not only the traumatic scene, but his younger self witnessing the event. He then walked around the body of his younger self to consider his facial expressions and his body posture. To do so, his perspective was turned away from the trauma scene and focused on his younger self. Similarly to Mr P, this allowed him to connect to new emotions of sadness and compassion for his younger self and alternative cognitions (e.g. ‘I can’t believe what I had to witness at that young age’).

Both clients appeared to benefit from this approach to place their fragmented images into a more dynamic 3D spatial memory. For Mr C, altering the spatial perspectives on the scene opened up the opportunity for greater toleration of his distress and subsequent updating through compassionate imagery techniques. For Mr P, manipulation of the scene allowed him to attend to aspects within the image that were previously unnoticed and thereby created a more embellished, contextualized memory, which then facilitated subsequent cognitive updating of the appraisals.

### Restructuring the narrative

The latter stage of therapy involved utilizing the imagined 3D scene, visualized now from multiple perspectives, to more freely facilitate the use of memory updating (Ehlers & Clark, 2000), via imagery rescripting techniques (Hackmann, 2011), to contextualize and process the fragmented memory. Following the spatial perspective shifts in the imaginal traumatic scenes, both clients began to access alternative cognitions that they used to restructure the narrative, e.g. for Mr C by utilizing compassion-focused methods. As with the previous stages, they continued to do this outdoors and rescripted the imaginal scenes with newly developed verbalizations or actions.

Mr P used a future perspective to consider where the bodies were now and why they needed to be at the morgue. As he walked around the imaginal scene, he was able to state new cognitions such as ‘this body needed to be at the morgue for identification purposes’ and ‘this allowed the man to get a proper funeral’. In doing so he was encouraged to focus on the new emotions that were elicited by these updated cognitions, which were more amenable to him than the initial feeling of horror that had overwhelmed him previously.

Mr C utilized the new feelings of compassion that had been elicited when viewing the past perspective of his younger self. He rescripted the imaginal scene by approaching his younger self, placing a hand on his imagined shoulder and stating ‘you’ve done well to handle this’ and ‘I’m so sorry you had to see this’. This allowed him to further connect with feelings of sadness and compassion, which in turn soothed him in the present day. Similar to Mr P this was a preferable and healthier emotion to access than the previously experienced feelings of horror and guilt.

## Treatment outcomes

Both Mr C and Mr P indicated positive outcomes post-treatment. They reported less anxiety and greater ability to focus on narrating their traumatic experiences while walking outdoors. Mr C attributed this to the benefits of the movement and the cool air temperature, while Mr P similarly benefited from the increased sensory grounding opportunities by focusing on the noise of road traffic. Both were therefore able to overcome their dissociation symptoms and engage in prolonged exposure, with increased habituation over time.

In terms of their intrusive imagery, both clients reported benefits from manipulating the perspectives of the traumatic scene. Mr P was able to see the images of the bodies as moved on in time, and to think of them as no longer in a morgue but within a formal burial. Mr C continued to re-experience his traumatic image of the explosion’s aftermath, but through readjusting the spatial properties and viewing the image from the past perspective, he was able to acknowledge and feel greater compassion towards his younger self, and in doing so his association with the traumatic image was improved.

From a clinician’s perspective, both clients were able to overcome the barriers of dissociation, the fragmented nature of their intrusive memories and to access the associated cognitions and update them with greater ease. This was achieved within the usual number of treatment sessions offered. Mr C and Mr P completed a number of outcome measures at the start and end of treatment. These included the PTSD Symptom Scale – Interview (PSS-I) for PTSD-specific symptoms. The maximum score on this measure is 51, with a cut-off score of 20 suggested as clinically significant for PTSD. We have found a 10-point reduction to be clinically significant (Murphy *et al.* 2015). The Work and Social Adjustment Scale (WSAS) was also used as a measure of wellbeing and quality of life, and the Dissociative Experiences Scale (DES) was included as a measure of dissociation.

Table 1 shows the pre-treatment and post-treatment scores for both Mr P and Mr C on these measures, indicating a meaningful reduction in scores on the PSS-I, a reduction in dissociative symptom scores, and improved functioning on the WSAS.

## Discussion

In this paper we discussed an adapted approach to imaginal reliving and prolonged exposure, as utilized in TF-CBT, with two main aims. First, to address high levels of dissociation that can prevent clients with PTSD from engaging in exposure work. Second, to address fragmented intrusive imagery, by shifting from a static spatial image to a richer spatial representation that includes allocentric frames of reference. In addition, cognitive restructuring focused on ‘moving’ the fragmented image around in time to support the transition to a more processed spatially and temporally dynamic memory. The key differences between this approach and traditional approaches to TF-CBT appear to be (1) a greater focus on multiple sensory grounding while engaging in prolonged exposure outdoors, (2) development of an imaginal 3D image of the trauma, and (3) including information about the spatial relationships between objects from multiple viewpoints. These are summarized in Table 2.

### Exploring the role of allocentric spatial processing

There is an emphasis on allocentric processing within this approach as it focused on making a 3D representation of the fragmented memory and exploring it from different perspectives. Neurobiological models of healthy visuo-spatial memory in humans (Burgess *et al.* 2002; Byrne *et al.* 2007) suggest that, during memory formation, perception drives activation of sensory cortices and input from the hippocampus and parahippocampal regions combine egocentric (viewpoint-dependent) perceptual representations with allocentric (viewpoint-independent) contextual representations. Over time, these low-level sensory representations (S-Reps) decay and become inaccessible unless reactivated via allocentric contextual representations (C-Reps), leaving a long-term memory that can generate egocentric imagery when deliberately retrieved.

A contemporary neurobiological model of intrusive memories [Revised Dual Representation Theory; DRT-R (Brewin *et al.* 2010)] suggests that under conditions of extreme stress due to trauma, allocentric processing is impaired and PTSD arises from disrupted encoding of the context of the event, relative to spared affective/sensory representations of the traumatic content. In this view, hippocampally mediated allocentric spatial representations form an important part of the contextual representations that are disrupted in PTSD (Brewin *et al.* 2010). Brewin and colleagues (2010) suggest that the formation of vivid sensation-based representations (S-Reps) without strong association to the corresponding contextual representations (C-Reps) allows S-Reps to intrude into the conscious in response to reminders. This gives rise to re-experiencing PTSD symptoms such as nightmares and flashbacks in which the emotions that were present at the time of the trauma appear to be re-created with their original intensity.

A recent study found that traumatized individuals with PTSD performed significantly worse on two tasks of allocentric spatial processing compared to trauma-exposed controls (Smith *et al.* 2015). These data suggest an underfunctioning in the part of the brain responsible for contextual representation of memories as predicted by the DRT-R (Brewin *et al.* 2010). With this in mind, it is suggested that techniques encouraging allocentric representations in the aftermath of trauma may hold a facilitative role in the activation of the hippocampus and the reduction in traumatic encoding (Smith *et al.* 2015). Therefore, in the current study, by asking the clients to imagine the scene of the trauma from different viewpoints, with a particular focus on the changes in spatial relationships of objects within the scene, this allowed us to encourage allocentric processing and facilitation of contextualization. We hypothesize that this adapted approach may also have helped clients to better manage their dissociation by allowing them to focus on less emotionally laden aspects of the scene, while also maintaining their presence within the imaginal reliving, and in doing so to be able to better tolerate their anxiety levels. However, to date we are not aware of any empirical data that supports this hypothesis.

### Limitations

There are important limitations to consider. This paper draws on a theory-driven approach and therefore requires empirical data to support these ideas. While studies have demonstrated a link between hippocampal size (Gilbertson *et al.* 2007) activation (Astur *et al.* 2006), allocentric spatial processing and severity of symptoms in sufferers of PTSD, the clinical applications of this link remain unclear. This paper is the first step in formalizing how reliving facilitation through allocentric spatial processing may be achieved. There is also a strong emphasis on managing dissociation throughout the paper, supported in part by allocentric processing as well as drawing from greater sensory grounding opportunities. We are aware the management of dissociation is not a new concept and is central to stabilization work prior to engagement with TF-CBT. However, we argue that traditional grounding methods within a clinic-room setting may benefit from adaption, particularly incorporating movement in a multi-sensory environment.

## Conclusions

In this paper we have suggested an adapted approach to TF-CBT that may be helpful for individuals with fragmented intrusive imagery with a strong visuo-spatial component. Through the use of two case studies we have described this adapted approach, considered the role of allocentric spatial processing and discussed the limitations. However, further systematic research is needed to determine the utility of this approach. A component analysis would elucidate which of the amendments to TF-CBT were effective or if it was a combination of both. Further investigation in populations with high levels of dissociation (e.g. survivors of torture and childhood trauma) would also be useful to determine the applicability of these amendments across client groups.

## Figures and Tables

**Table 1:** Pre-admission, admission and post-admission scores for Mr C and Mr P

	Admission	Discharge
Mr C		
PSS-I	33	23
WSAS	32	14
DES	19	10
Mr P		
PSS-I	41	19
WSAS	33	25
DES	49	30

PSSI, Post-traumatic Stress Symptom – Inventory; WSAS, Work and Social Adjustment Scale; DES, Dissociative Experiences Scale.

**Table 2:** Similarities and differences in approach to traditional TF-CBT methods

Stage of treatment	Similarities	Differences
Walking through the narrative	Stabilizing client using grounding to manage anxiety	Greater focus on multiple sensory grounding for highly dissociative clients, e.g. movement, air temperature, textures, noise
	‘*In vivo*’ exposure to the trauma memory	Greater focus on developing a 3D spatial plan of the image that the client can walk around
Manipulating the perspectives	Challenging cognitions and developing alternative cognitions	Considers the trauma image from multiple viewpoints to readjust the spatial relationships between objects
		Develops the contextual factors to create a dynamic memory rather than fragmented image
Restructuring the narrative	Cognitive restructuring and updating of the memory	None

## Summary of key points

(1) Some clients with PTSD can face a number of difficulties when engaging with traditional approaches to imaginal reliving and prolonged exposure, utilized within the framework of TF-CBT. These include high levels of avoidance, dissociation and fixed ‘viewpoint-dependent’ intrusive images.
(2) In this paper we have found that adapting imaginal exposure methods to have a stronger emphasis on grounding and allocentric processing may be helpful for a subset of patients with PTSD that present with high levels of dissociation and are highly avoidant.
(3) We acknowledge the limitations of a case study paper and suggest that further research is needed to compare this adapted approach with traditional approaches to TF-CBT.

## Learning objectives

(1) To consider some of the barriers that a subset of traumatized clients may present with, when using traditional methods of imaginal reliving and cognitive updating in TF-CBT.
(2) To learn about an adapted approach which may be helpful for clients who struggle with high levels of dissociation, avoidance, and fixed intrusive images.
(3) To learn how this can approach can enhance traditional TF-CBT techniques, alongside imagery rescripting and third-wave approaches.
(4) To learn about the theoretical hypotheses to this adapted approach, using allocentric processing.
